# Improving the Electrical Percolating Network of Carbonaceous Slurries by Superconcentrated Electrolytes: An Electrochemical Impedance Spectroscopy Study

**DOI:** 10.1021/acsami.1c02439

**Published:** 2021-03-10

**Authors:** Alessandro Brilloni, Federico Poli, Giovanni Emanuele Spina, Damiano Genovese, Giorgia Pagnotta, Francesca Soavi

**Affiliations:** † Department of Chemistry “Giacomo Ciamician”, Alma Mater Studiorum Università di Bologna, Via Selmi 2, Bologna 40126, Italy; ‡ Bettery Srl, Via Pisacane 56, Massafra 74016, Italy

**Keywords:** semisolid slurry, semisolid redox flow battery, electrical percolating network, superconcentrated electrolyte, electrochemical impedance spectroscopy, optical fluorescence microscopy, semisolid slurry viscosity

## Abstract

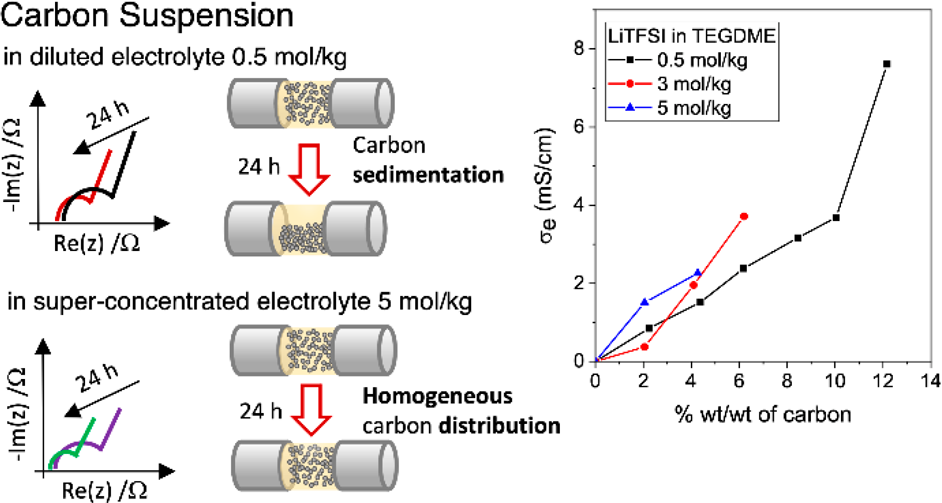

Semisolid redox flow batteries simultaneously address the need for high energy density and design flexibility. The electrical percolating network and electrochemical stability of the flowable electrodes are key features that are required to fully exploit the chemistry of the semisolid slurries. Superconcentrated electrolytes are getting much attention for their wide electrochemical stability window that can be exploited to design high-voltage batteries. Here, we report on the effect of the ion concentration of superconcentrated electrolytes on the electronic percolating network of carbonaceous slurries. Slurries based on different concentrations of lithium bis(trifluoromethane)sulfonamide in tetraethylene glycol dimethyl ether (0.5, 3, and 5 mol/kg) at different content of Pureblack carbon (from 2 up to 12 wt %) have been investigated. The study was carried out by coupling electrochemical impedance spectroscopy (EIS), optical fluorescence microscopy, and rheological measurements. A model that describes the complexity and heterogeneity of the semisolid fluids by multiple conductive branches is also proposed. For the first time, to the best of our knowledge, we demonstrate that besides their recognized high electrochemical stability, superconcentrated electrolytes enable more stable and electronically conductive slurry. Indeed, the high ionic strength of the superconcentrated solution shields interparticle interactions and enables better carbon dispersion and connections.

## 1 Introduction

Boosting the shift to a low carbon society requires a large and widespread diffusion of renewable energy sources such as solar, wind power or ocean wave energy. However, all these sources connected to the power grid are intermittent and can be only partially forecasted. For this reason, the energy network necessitates the development of large electrical energy storage (EES) systems that level loads, shave peaks and improve the overall plant efficiency. One of the most suitable and used EES technology for this purpose is represented by redox flow batteries (RFBs).^1,2^ An RFB is a rechargeable battery that stores electrical energy in two different soluble redox couples. The liquid components are stored in external tanks and are pumped through the reactor, where the reaction and the consequent ion exchange occurs. Therefore, the total capacity and energy of the RFB are related to the volume of the reactants contained in the tanks. Because the active species are dissolved in the electrolyte, the higher is the solubility of the active material, the greater is the capacity and energy densities. The total power output, instead, is determined by the electrode plate area inside the stack and by the design of the reactor. The main advantage of RFBs is the high flexibility in meeting the demand of the final user. Indeed, energy and power are effectively decoupled. The most diffused technology for RFBs are the vanadium redox flow battery (VRFBs). Despite the long cycle life (>10000 cycles, 10/20 years), these RFBs suffer from low operational potential (<2 V), low energy density, and narrow duty temperature range.^2−4^

These constraints are pushing researchers to look for new redox couples and designs of RFBs. The main strategies that have been proposed to increase energy and power of RFBs include the use of: (i) organic electrolytes to increase the cell voltage above 2 V and widen the temperature operation, (ii) solid metal anodes, and (iii) sulfur- or O_2_ (air)-based catholytes to reduce volume and weight, and (iv) semisolid anolyte and/or catholyte to circumvent the active materials solubility limitation of conventional RFBs.^2,5,6^ In the last case, because the redox couples are not molecules in solution but solid particles suspended in the electrolyte, the active species will not crossover through the separator. Ion conduction will take place through the porous voids of the separator, approaching the electrolyte bulk conductivity with advantages in terms of power. However, the development of semisolid RFBs requires efficient management of the viscous slurries that results from the particle suspension, a smart management of the slurry flow, and improved reactor design.^7^ A combination of these different approaches has led to many RFB configurations.^2−6,8^ Gogotsi et al. proposed a new concept of supercapacitor called the “electrochemical flow capacitor” (EFC) that benefits from the major advantages of both supercapacitors and flow batteries.^9^ RFBs featuring semisolid catholytes based on LiCoO_2_ (LCO) or LiMn_1.5_Ni_0.5_O_4_ (LMNO) or semisolid anolytes with Li_4_Ti_5_O_12_ (LTO) or LiFePO_4_ (LFP) were also studied.^10−14^ The highest specific energy of 500 Wh/kg was demonstrated by combining a lithium metal anode and a semisolid O_2_ catholyte made of a suspension of carbon (2%) in a solution of 0.5 *m* solution of lithium bis (trifluoromethane)sulfonamide (LiTFSI) in tetraethylene glycol dimethyl ether (TEGDME) saturated with O_2_.^15,16^

Nowadays, the most challenging issues in the development of high-specific-energy RFBs are related to (i) the formulation of carbonaceous conductive slurries with an efficient electrical percolation network; (ii) the use of stable organic electrolytes to minimize the side reactions with electrolytes and the other cell components, such has the current collectors; and (iii) the achievement of high cell voltages. An efficient electrical percolation network is required to improve the kinetics of the faradaic processes, and, specifically, the electron transfer between the particles and the current collector. The consequence is that ohmic losses are minimized and the power is improved. The rheological and conductive behaviors of the slurries depend on carbon morphology and surface chemistry, which in turn affect particle agglomeration/sedimentation in the electrolyte solution.^3,13,17−21^ In our previous work, we reported that by the proper selection of the carbon, it is possible to formulate slurries with high carbon content characterized by good electrical and rheological properties. Indeed, we compared the electrochemical response of carbonaceous catholytes of semisolid Li/O_2_ flowable battery based on different weight percents of Super-P (spherical particles) and Pureblack (fragmental particles) carbons in 0.5 *m* LiTFSI in TEGDME. According to our findings, the fragmental morphology of Pureblackparticles enabled the formulation of pseudoplastic slurries capable of reducing their viscosity at increased share rate even at high carbon content. In turn, these features positively affected energy and rate performance of the catholyte. In addition, a smooth flow is expected to lower the power consumption of the pump.^7,14^

Regarding the electrolyte, its composition has to be properly formulated, especially when high-voltage semisolid RFB operation is targeted. In RFBs that exploit the lithium battery chemistries, the solid electrolyte interface (SEI) formation in fluid electrodes represents a critical issue. Indeed, the electronic insulator nature of SEI hinders the electrical contact between the current collector and the particles dispersed in the electrolyte. The use of ionic liquids and superconcentrated (solvent-in-salt) electrolytes with their recognized wide electrochemical stability window, might represent a solution.^22^ Superconcentrated solutions of LiTFSI in TEGDME are gaining much interest as a new class of stable electrolytes.^23^ Furthermore, they represent an extremely interesting platform for the study of the effect of the electrolyte “structure” in terms of ion interactions on the electrochemistry of the systems. Indeed, the increase in LiTFSI concentration from conventional values up to its maximum value, i.e., ca. 5 mol kg^–1^ at room temperature, modifies the electrolyte behavior from that of a classical salt-in-solvent solution to a solvent-in-salt solution. Increasing LiTFSI concentration from 0.5 mol kg^–1^ to 5 mol kg^–1^, changes conductivity from ca. 2 to 0.7 mS cm^–1^.^24^ However, the 5 *m* solution of LITFSI-TEGDME features an extremely high viscosity of 550 cP that might prevent its use as flowable electrolyte. Therefore, a slightly lower concentration of LITFSI in TEGDME, such as 3 *m*, could represent a good balance between good electrochemical stability, conductivity, and fluidity.^24^

Here, for the first time to our knowledge, we investigate the effect of the electrolyte ion concentration, from conventional to superconcentrated, on the electronic percolating network of carbonaceous slurries for lithium-ion semisolid RFBs. Specifically, this study mainly focuses on three different concentrations of LiTFSI in TEGDME, namely 0.5, 3, and 5 molal, at different content of Pureblack carbon. The electrical properties of conductive slurries and their dynamic behavior were investigated by a deep electrochemical impedance spectroscopy (EIS) analysis complemented by optical fluorescence microscopy and rheological studies. A model that describes the complexity and heterogeneity of the semisolid fluids by multiple conductive branches of an equivalent circuit model, is also proposed. Our study demonstrates that superconcentrated electrolytes have a structuring effect on the distribution of carbon particles which positively affects the electronic percolation network of the semisolid slurries of RFBs.

## 2 Experimental Section

### 2.1 Materials

Two different electrolyte compositions were used to formulate slurries. Tetra-ethylene-glycol-dimethyl-ether (TEGDME, ≥ 99%) and lithium bis-tri-fluoro-methan-sulfonimide (LiTFSI, ≥ 99%) were purchased by Sigma-Aldrich and used to prepare the electrolytes. The electrolyte 0.5ME featured a LiTFSI molal concentration equal to 0.5 mol kg^–1^. For the electrolytes, 3ME and 5ME the LiTFSI molal concentrations were 3 and 5 mol kg^–1^. Conductivity, density, and dynamic viscosity of 0.5ME, 3ME, and 5ME were 2.2 mS cm^–1^ (at 30 ± 0.3 °C), 1.08 g cm^–3^, 7.14 cP, 2.0 mS cm^–1^ (at 30 ± 0.3 °C),1.31 g cm^–3^, 47.1 cP, and 0.8 mS cm^–1^ (at 30 ± 0.3 °C), 1.43 g cm^–3^, 550 cP, respectively.^24^ The carbon used to compose the slurry was Pureblack 315 (PB), (BET 64 m^2^ g^–1^) from Superior Graphite. The carbonaceous particles were dispersed at different percentages in the electrolyte solutions. All the slurries were prepared inside a drybox (MBraun LabmasterSP 130) with an argon atmosphere (H_2_O < 0.1 ppm, O_2_ < 0.1 ppm). Before use, LiTFSI was dried in an electric oven at 120 °C for 12 h under a dynamic vacuum. PB carbon was dried in the same oven at 120 °C for 12 h and both components were stored in the drybox. Table 1 reports the identification codes of the slurries along with their composition in terms of carbon mass percentage and of the ratio of the carbon mass to the electrolyte volume. In the case of the electrolyte 0.5ME we explored a carbon content ranging from ∼2 to 12 wt %. For the 3ME electrolyte, the maximum feasible carbon content that enabled a fluid slurry was 6 wt %. For the 5ME, it was not possible to add more than 4 wt % carbon for the same reason.

**Table 1:** Slurry Identification Code and Composition in Terms of Carbon Mass Percentage and of the Ratio of Carbon Mass to the Electrolyte Volume

0.5ME-based slurry (0.5 mol kg^–1^ LiTFSI)						
sample ID code	PB052	PB054	PB056	PB058	PB0510	PB0512
%C (w/w)	2.23	4.38	6.17	8.3	10.04	12.17
C content (g cm^–3^)	0.025	0.049	0.071	0.098	0.121	0.150
3ME-based slurry (3mol kg^–1^ LiTFSI)						
sample ID code	PB32	PB34	PB36			
%C (w/w)	2.05	4.1	6.2			
C content (g cm^–3^)	0.027	0.056	0.086			
5ME-based slurry (5mol kg^–1^ LiTFSI)						
sample ID code	PB52	PB54				
%C (w/w)	2.5	4.2				
C content (g cm-3)	0.029	0.063				

### 2.2 Optical Fluorescence microscopy

To better highlight the arrangement of the carbon agglomerates, we collected optical fluorescence microscopy images of the slurries. The instrument was an Olympus IX71 with 10× objective lens. [9-(2-carboxyphenyl)-6-diethylamino-3-xanthenylidene]-diethylammonium chloride (rhodamine B) was chosen as dye because it can be dissolved, giving fluorescence and making an easier study of the carbon dispersions feasible. For this purpose, 0.1μg of dye was added to 100 μg of slurry.

### 2.3 Electrochemical Impedance Spectroscopy

Electrochemical Impedance measurements were performed by using T-shaped cells with two stainless steel blocking electrodes (Figure S1) (1 cm diameter) at ∼1 cm of distance. The EIS spectra were collected with a BioLogic VSP multichannel potentiostat/galvanostat/FRA within a 50 kHz to 1 Hz frequency range and 10 mV of perturbation amplitude, acquiring 10 points per decade (selected frequencies ranges are specified when required). EIS spectra collected in a wider frequency range (200 kHz to 100 mHz) are reported in Figure S2. To guarantee the reproducibility of the measurements, we defined a specific procedure for testing. First, we stirred the samples to be tested for 20 min and they were then transferred inside the glovebox to assemble the cells that were previously heated at 30 °C. When assembled, the cells were stored in a thermostatic oven at 30 °C and EIS measurements were performed over time. Specifically, two measurements were carried out immediately after cell assembly in sequence (identified as “-01” and “-02”). The test was repeated after half an hour (identified as “-03”), and the last one after an additional 24 h (identified as “-04”). The overall test spanned for a time of 24.5 h.

### 2.4 Electrochemical Impedance Spectroscopy Analysis

**Figure 1:**
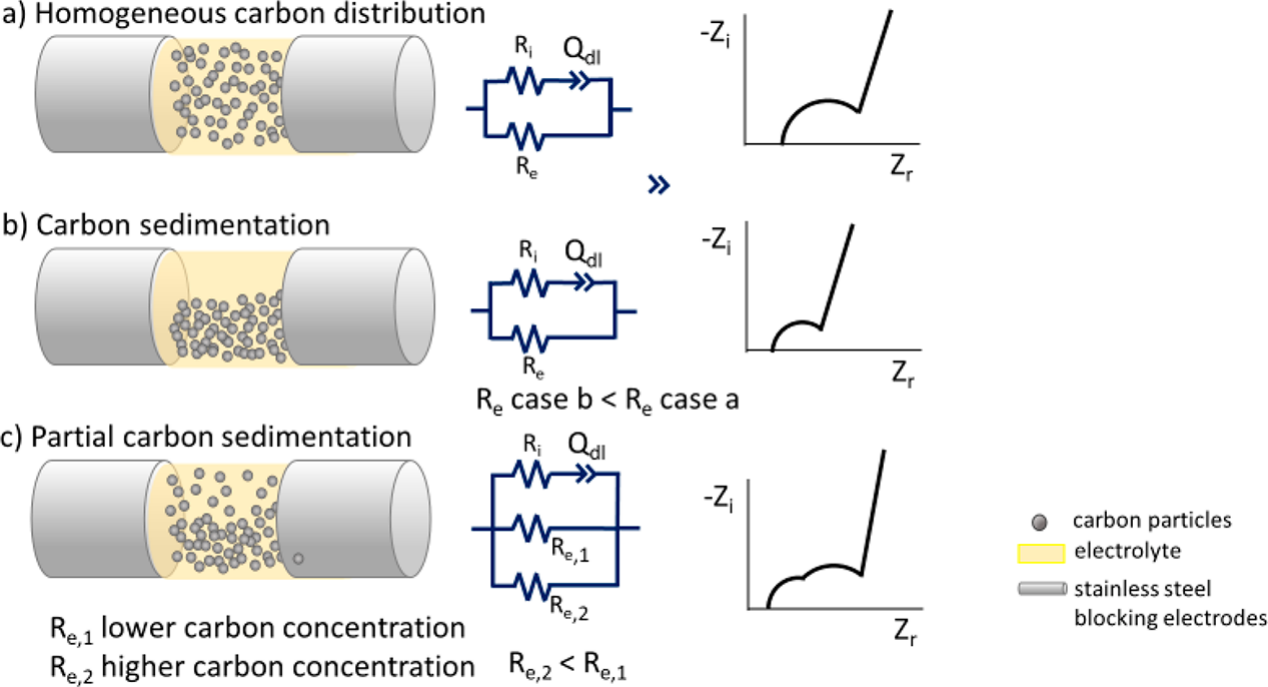
Dispersion of the carbonaceous slurry between the two stainless steel blocking electrodes, the equivalent circuit that models the electrical response of the cell and the corresponding ideal Nyquist plot for (a) homogeneous carbon dispersion, (b) carbon completely sedimented on the bottom of the cell, and (c) partially sedimented.

The EIS spectra of the carbonaceous suspensions were analyzed referring to the model proposed by Youssry et al.^17^ Specifically, the impedances of the suspensions have been modeled with the equivalent circuits reported in Figure 1a.

Figure 1a describes the homogeneous dispersion of the carbonaceous slurry between the two stainless steel blocking electrodes, the equivalent circuit that models the electrical response of the cell and the corresponding ideal Nyquist plot. *R*_e_ represents the electronic resistance of the dispersed carbon particles, in parallel with the ionic branch *R*_i_*Q*_dl_. *R*_i_ is the ionic resistance of the electrolyte, which is in series with the constant phase element *Q*_dl_. The latter represents the electrical double-layer capacitance at the blocking electrode/slurry interfaces. When the concentration of carbon in the slurry is extremely low and *R*_e_ is much higher than *R*_i_, the *R*_e_ branch of Figure 1a is negligible. In this case, the electrochemical response of the cell filled with the slurry corresponds to that of an electrochemical capacitor. At high frequencies (e.g., 50 kHz), the real component of the impedance mainly corresponds to the ionic resistance *R*_i_. At low frequencies, the impedance is dominated by the capacitive accumulation of ions at the surface of the blocking electrodes. Therefore, the corresponding Nyquist plot approximates a straight line parallel to the imaginary axis, with intercepts at high frequencies on the real axis at *R*_i_. When the concentration of carbon is sufficiently high, a good electronic percolating network is set and the *R*_e_ branch becomes relevant. Given the high number of carbonaceous particles in contact with each other, at low frequencies, an electronic current through *R*_e_ is also observed in parallel with the ionic one associated with the formation of the double layer and that flows through the *R*_i_*Q*_dl_ branch. Consequently, the Nyquist plot changes from a straight line to a semicircle. The real axis intercept at high frequencies *R*_hf_ includes ionic and electronic terms according to the equation:
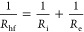
1The electronic conductivity of the slurries, σ_e_, can be evaluated as reciprocal of *R*_e_
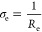
2where
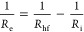
3In Figure 1a, the semicircle diameter quantifies the resistance of the electronic percolating network *R*_pn_. Agglomeration and sedimentation phenomena can give rise to a dynamic electrical response of the slurry. Indeed, Figure 1b represents the case of a slurry where the carbon is sedimented. After sedimentation, carbon particles form aggregates and become better connected. Consequently, *R*_hf_ and *R*_e_ decrease, and the semicircle shrinks and shifts to the left of the Nyquist plot. Hence, it is worth noting that the carbonaceous percolating network should rather be described as a complex system composed of different branches that have different connection paths. The different branches correspond to portions of the slurry that feature different carbon concentration and aggregations status. This is described by Figure 1c, which models a slurry with carbon partially sedimented. In this case, the equivalent circuit is modified by adding different *R*_e_ branches that are in parallel with the ionic one. As an example, in Figure 1c, only an additional branch *R*_e,2_ is added. Therefore, the corresponding Nyquist plot will feature two semicircles, each one representing the two different percolating networks.

### 2.5 Rheological Measurements

The viscosity of the slurries was evaluated by using Anton Paar MCR 102 rheometer in a plate–plate geometry with a diameter of 25 mm and a gap of 0.5 mm. The measurements were carried out with a shear rate range from 0.01 to 1000 s^–1^ by keeping the temperature constant at 25 °C.

## 3 Results and Discussion

The effect of carbon content and electrolyte concentration on the percolating network of the semisolid slurries was evaluated by EIS. Specifically, three different electrolyte concentrations were explored, i.e., 0.5 molal (0.5ME), 3 molal (3ME), and 5 molal (5ME). The next Subsections report the studies carried for each electrolyte composition. Section 3.1 refers to 0.5ME-based slurries, section 3.2 to samples with 3ME electrolyte, section 3.3 compares the results obtained with the different electrolytes formulations, including the 5ME, and finally, section 3.4 reports and discusses the rheological features of the slurries.

### 3.1 Slurries with 0.5ME Electrolyte

**Figure 2:**
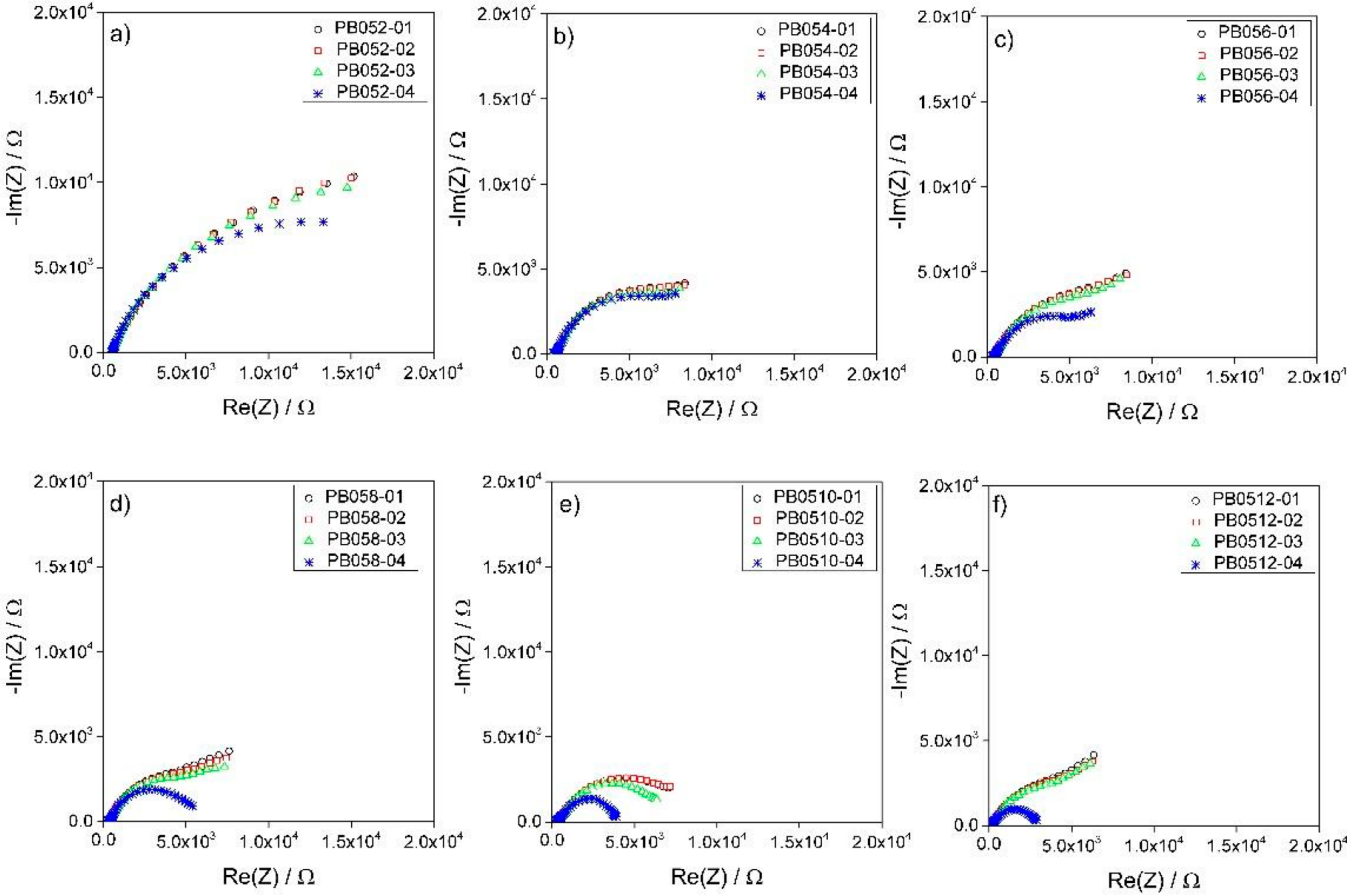
Nyquist plots evolution over time of 0.5ME-based slurries with different carbon percentages of (a) 2, (b) 4, (c) 6, (d) 8, (e) 10, and (f) 12% from 50 kHz to 1 Hz.

**Figure 3:**
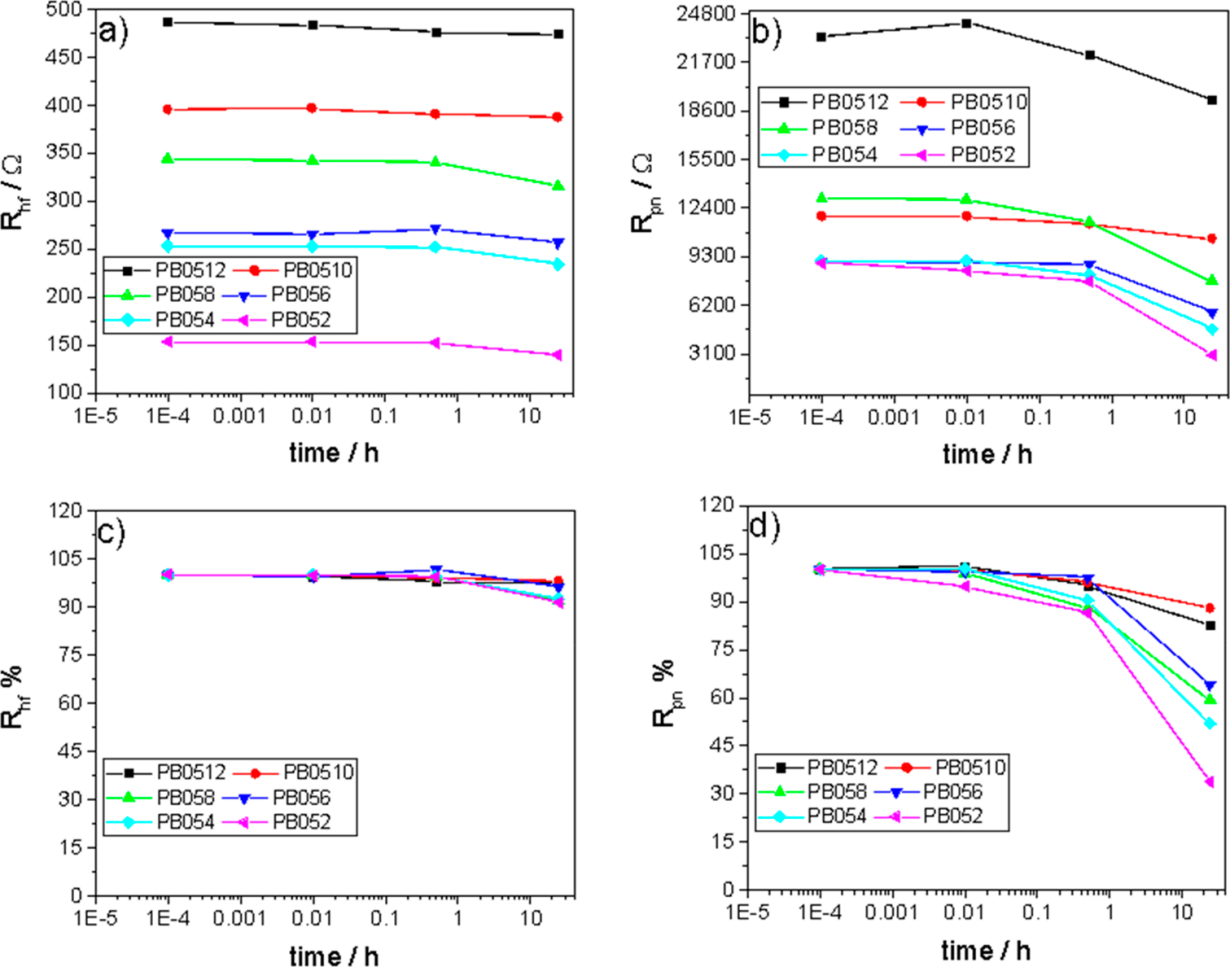
Evolution over time of (a) the high frequency resistance (*R*_hf_) and (b) the percolating network resistance (*R*_pn_) of 0.5ME-based slurries at different carbon percentages, and of their quantities (c) *R*_hf_ % and (d) *R*_pn_% normalized by their corresponding initial values.

In this section, carbonaceous suspensions with the electrolyte 0.5ME (0.5 molal) at different carbon contents are investigated. Table 1 details the composition of the investigated samples containing a carbon weight percentage ranging from 2.23 to 12.17%. At first, EIS was used to monitor the evolution of the slurry impedance over time. Figure 2 reports the Nyquist plots in the frequency range 50 kHz to 1 Hz for each 0.5ME-based sample, collected immediately after cell assembly (data labeled with ”-01” and “-02” were made consequentially), after 30 min (data labeled “-03”), and after 24 h from the last measure (data labeled “-04”). All the plots are representative of slurries featuring both ionic and electronic conductivity. Indeed, as mentioned in section 2.4., they feature large semicircles. The semicircles have a high-frequency intercept on the real axis (*R*_hf_) that is related to electronic and ionic resistances and a diameter *(*R_pn_) that depends on the efficiency of the carbonaceous percolating network. All the samples show a strong time dependence of the impedance spectra over time. Furthermore, the semicircles tend to shrink with the increase of the carbon content. As it concerns the time evolution, for each sample, the two initial measures, -01 and -02, show no significant difference and they almost overlap. After 30 min and, mainly, after 24 h, a strong decrease of the semicircle diameter, and therefore, of the percolating network resistance *R*_pn_, is noticeable. In parallel, the semicircles shift to lower *R*_hf_ values. This is highlighted by Table S1 and Figure 3a and Figure 3b, which report the values of *R*_hf_ evaluated at 50 kHz and of *R*_pn_ evaluated over time. The latter was calculated by analyzing the Nyquist plot by a nonlinear fitting procedure referring to the equivalent circuit shown in Figure 1 and discussed in section 2.4. Figure 3c, d reports the values of *R*_hf_ and *R*_pn_ normalized with respect to their initial values, i.e., to the values recorded immediately after cell assembly. *R*_pn_ changed from 23.3 to 19.3 kΩ (82.8%) for PB052, from 11.8 to 10.4 kΩ (88.1%) for PB054, from 13.3 to 6.8 kΩ (51.1%) for PB056, from 9 to 5.7 kΩ (63.3%) for PB058, from 8.9 to 4.6 kΩ (51.7%) for PB0510, and from 8.8 to 3 kΩ (34.1%) for PB0512 (Figure 3b, d). Even for *R*_pn_, the percentage variation over time was more evident at high carbon content: for PB052, the *R*_pn_ decreased by 17%, and for PB0512, *R*_pn_ decreased by 66%.

Overall, the data reported in this section demonstrate that (i) the increase in carbon content effectively reduces the resistance of the slurries, and (ii) the slurries have dynamic behavior, which changes depending on the carbon content.

**Figure 4:**
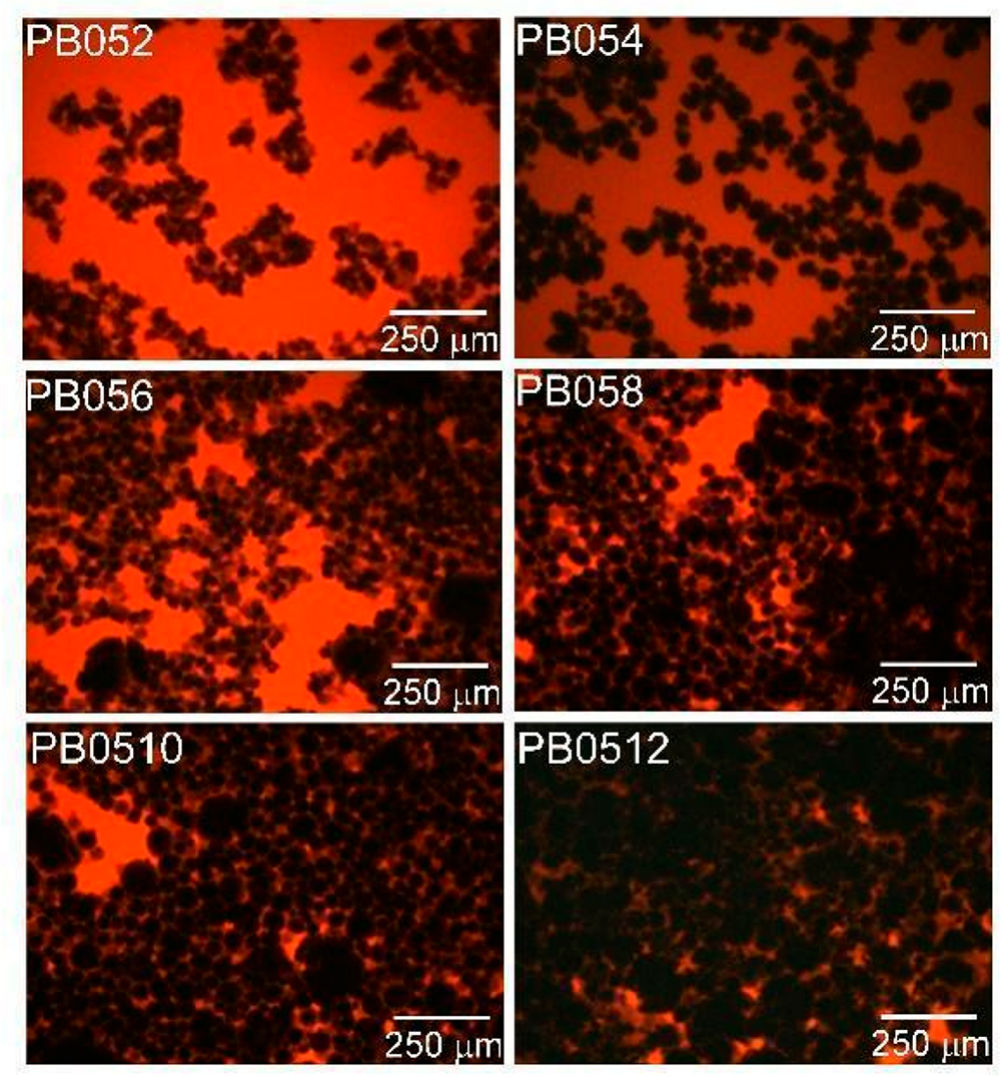
Optical fluorescence images of 0.5ME-based slurries with different carbon content.

Figure 4 reports the optical fluorescence images of 0.5ME-based slurries with different carbon percentages and clearly shows that the number of carbon particles electrically connected increases when moving from PB052 to PB0512. The images show that the samples with the higher carbon concentration display a better connection among the carbon particles, which are more inclined to produce an efficient percolating network. This explains the observed trends of the Nyquist diagrams that show a constant reduction in impedance with an increase in carbon content. Figure 4 even suggests that, at high carbon content, the electrolyte is not able to shield the surface interactions among the carbon particles that, as a consequence, exhibit a higher tendency to agglomerate with respect to low carbon content samples. In turn, for the 0.5ME-based slurries, the increase in carbon content accelerate the impedance evolution over time toward lower values. Therefore, this behavior can be explained by the sedimentation or agglomeration of carbon particles in the liquid phase. This process gives rise to additional percolating network branches in parallel to the initial ones. The new branches are generated by the portion of the slurries that feature higher concentration of carbon, and, hence, a lower local *R*_pn_. This concept is described by Figure 1, which compares the electric behavior of an homogeneous slurry with carbon particles well distributed within the liquid phase and of a nonhomogeneous slurry with carbon particles totally or partially agglomerated in the bottom part of the fluid. This is further highlighted by the Nyquist plots of the samples PB052 and PB0512, collected in a wider frequency range (200 kHz to 100 mHz) and shown as an example in Figure S2. The plots indicate the presence of two semicircles that we relate to the presence of two different percolation branches. This is more evident for the slurry PB0512, which has the highest carbon content.

### 3.2 Slurries with 3ME Electrolyte

**Figure 5:**
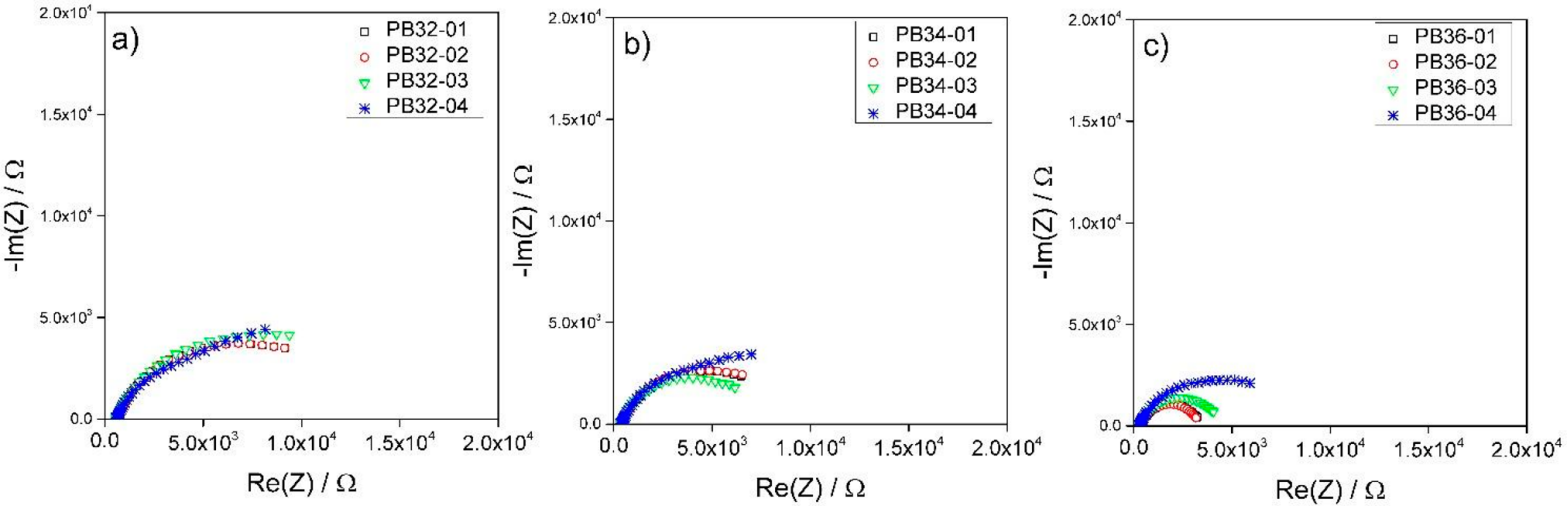
Nyquist plot evolution over time of 3ME-based slurries with different carbon percentages of (a) 2, (b) 4, and (c) 6% from 50 kHz to 1 Hz.

**Figure 6:**
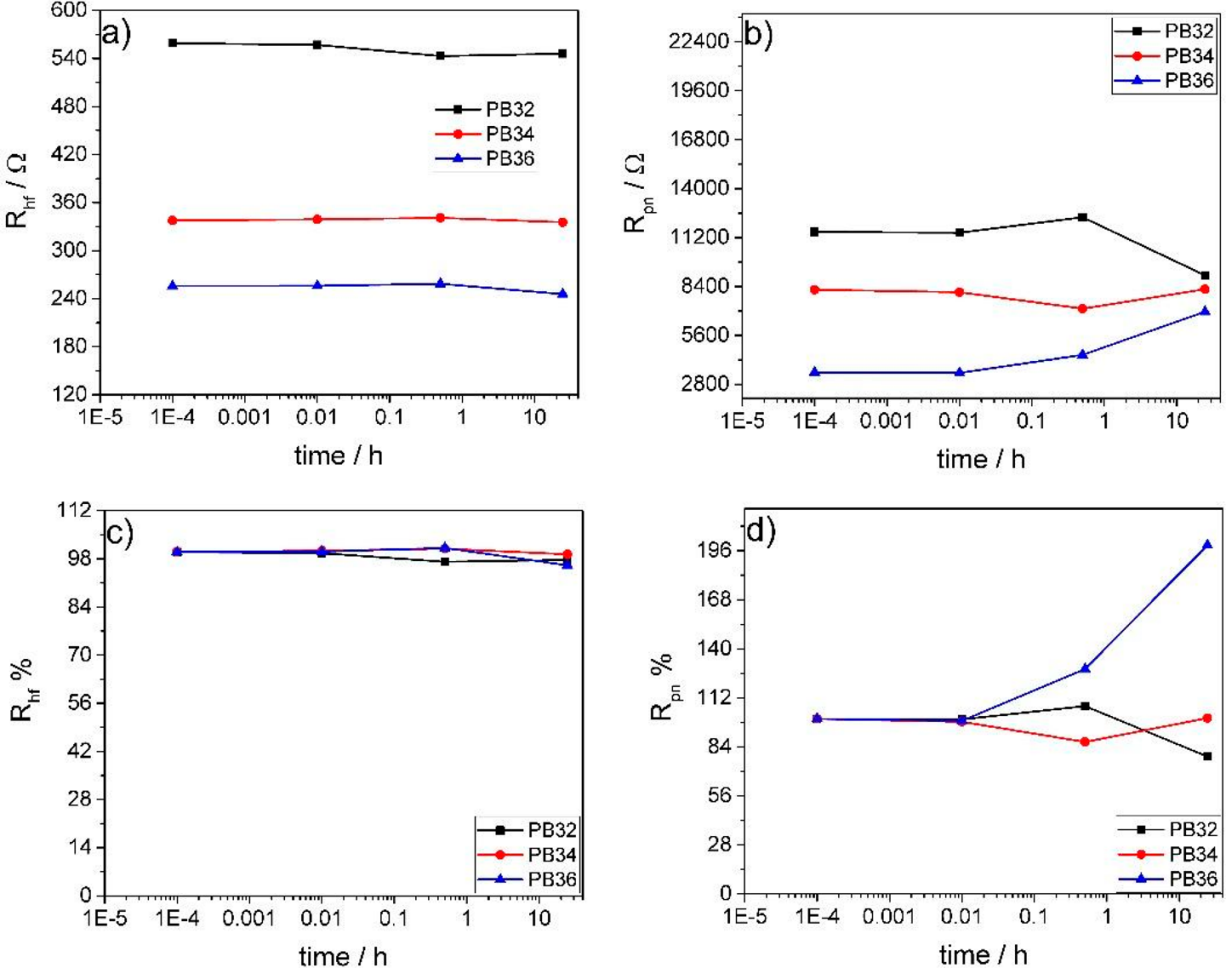
Evolution over time of (a) the high frequency resistance (*R*_hf_) and (b) the percolating network resistance (*R*_pn_) of 3ME-based slurries at different carbon percentages, and of their quantities (c) *R*_hf_ % and (d) *R*_pn_ % normalized by their corresponding initial values.

This section reports the EIS study of the slurries PB32, PB34, and PB36 that are based on the same superconcentrated electrolyte (3 *m* LiTFSI in TEGDME) but feature increasing carbon content from 2.05 to 4.1 and 6.2%. It is worth noting that with 3ME electrolyte, it was not possible to obtain a homogeneous slurry with a mass percentage of carbon greater than 6.2% because the fluid was too viscous. EIS measurements were carried out and analyzed following the same approach used for 0.5ME carbon slurries. Figure 5 shows the Nyquist plots collected immediately after the cell assembly (data labeled with ”-01” and “-02” were made consequentially), after 30 min (data labeled “-03”), and after 24 h from the last measure (data labeled “-04”). Even for 3ME samples, the Nyquist plots are representative of slurries featuring both ionic and electronic conductivity and evolve with a strong dependence on carbon content. The analysis of the Nyquist plots of Figure 5 provided the values of *R*_hf_ evaluated at 50 kHz and of *R*_pn_ that are plotted over time in Figure 6a, b and given in Table S2. Figure 6c, d reports *R*_hf_ and *R*_pn_ normalized to their initial values. Figure 5 shows that for each sample, the two initial measures -01 and -02 overlap. After 30 min (measure -03), it is not possible to appreciate a clear trend. Indeed, for PB32 and B36, the semicircle slightly widens. For PB34, the semicircle instead shrinks. However, after 24.5 h (measure -04), the Nyquist plots of all the samples seem to evolve into two semicircles that are representative of two different percolating network branches. Given that the two semicircles are not well resolved, *R*_pn_ was calculated by taking into account only the data in the highest frequency range cut at 8 Hz and excluding those at the lowest frequency. Therefore, the second semicircle was not considered. *R*_hf_ was found to slightly decrease for all the samples. Indeed, during the 24.5 h, it changed from 559 to 546 Ω (97.7%) for PB32, from 338 to 335 Ω (99.1%) for PB34 and, from 256 to 245 Ω (95.7%) for PB36 (Figure 6, c). The percentage decrease in *R*_hf_ was 2.3, 0.7, and 3.9% for PB32, PB34, and PB36, respectively. After 24.5 h, *R*_pn_ of PB32 slightly decreased from 11.5 to 9 kΩ (78.3%), therefore by 22%. For PB34, *R*_pn_ was instead almost constant at 8.2 kΩ, whereas for PB36, *R*_pn_ doubled from 3.5 to 7 kΩ (200%) (Figure 6b, d). The comparison of the data reported in this section with those in section 3.1 unveils a specific effect of the electrolyte composition on the resulting impedance of the slurries that will be further discussed in section 3.3.

**Figure 7:**
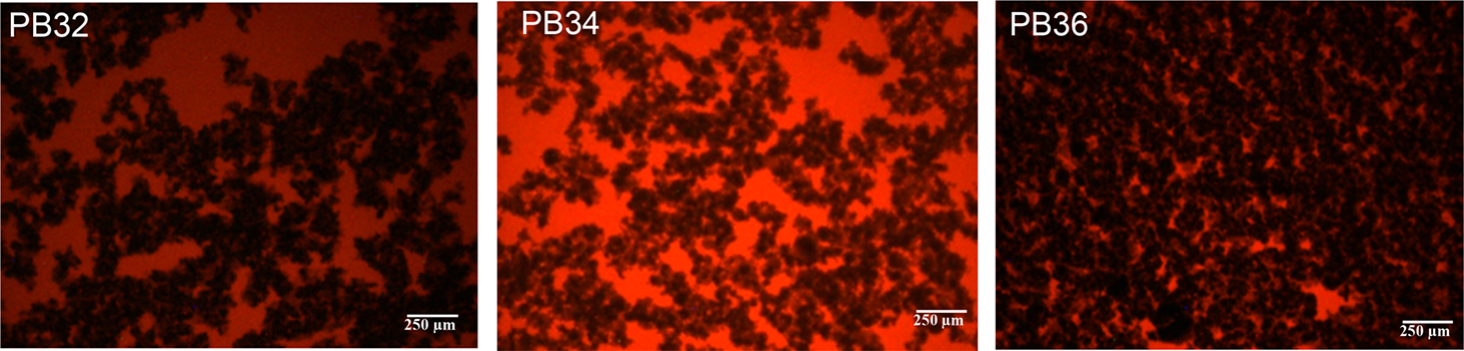
Optical fluorescence images of 3ME-based slurries with different carbon percentage.

The main difference is that the *R*_pn_ values of the PB32 and PB34 slurries are almost 50 and 20% smaller than those of 0.5ME-based samples at the same carbon percentage of 3 and 4%. This indicates that electrical percolation is more efficient when the 3ME electrolyte is used. The ionic strength of the superconcentrated solution might shield interparticle interactions, therefore enabling a better carbon dispersion and electronic connection. This is supported by the fluorescence images of samples PB32, PB34, and PB36 that are reported in Figure 7.

A second effect is that 3ME mitigates the evolution of the Nyquist plots and *R*_hf_ and *R*_pn_ over time. Hence, highly concentrated electrolytes are beneficial for the stabilization of carbonaceous slurry. In 3ME samples, a second semicircle at low frequencies appears after 24 h. This can be explained with the presence of the sedimentation and aggregation processes. Indeed, 3ME fresh samples feature a good percolating network with only one semicircle. After 24 h, sedimentation and agglomeration of carbon provide two branches. The first semicircle at higher frequencies is given by the concentrated portion of agglomerated particles (low *R*_pn_). The second one, at lower frequencies, is related to diluted carbons particles that are still suspended in solution (high *R*_pn_).

### 3.3 Comparison of 0.5ME-, 3ME-, and 5ME-Based Slurries

**Figure 8:**
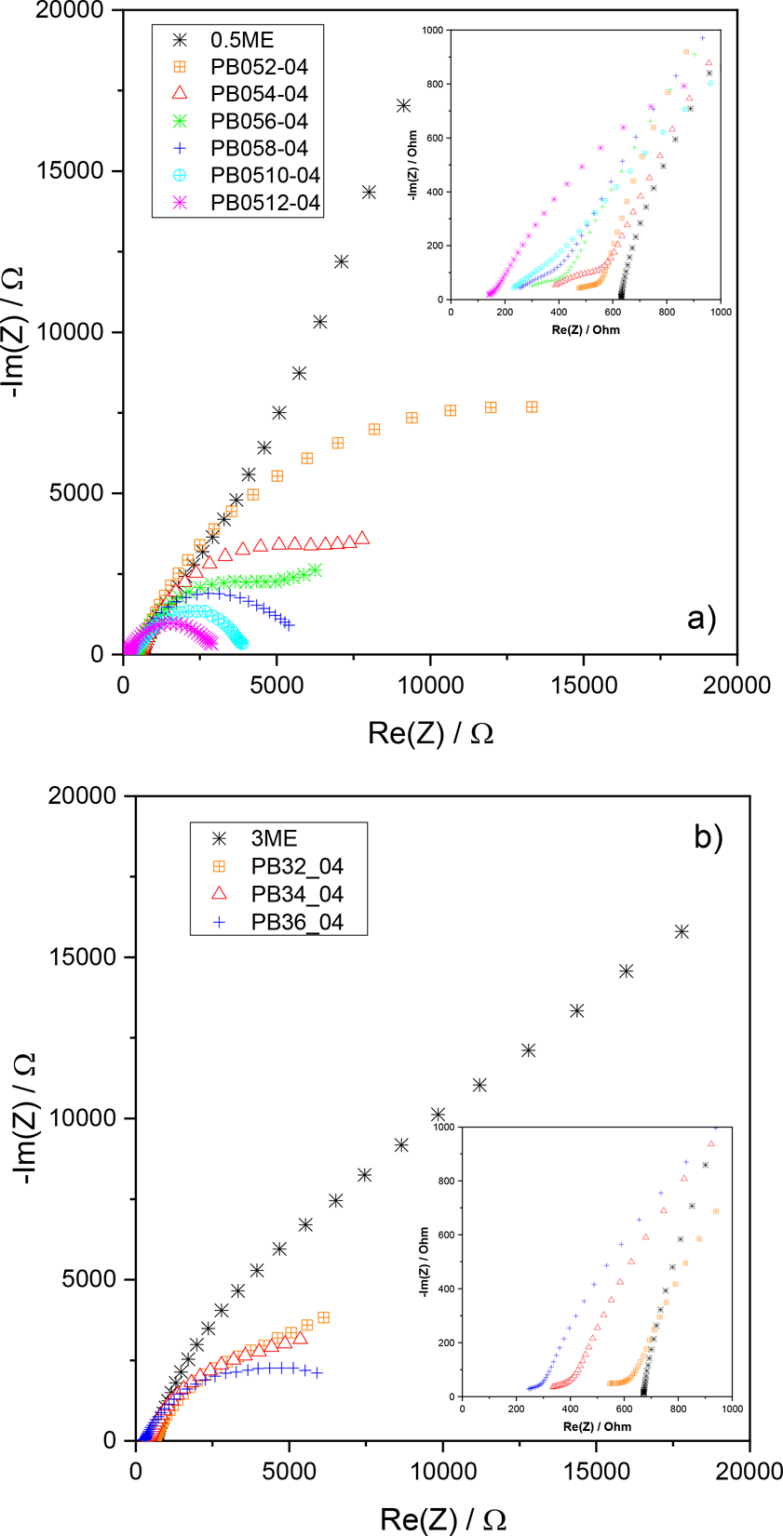
Comparison of the Nyquist plots collected from 50 kHz to 1 Hz, after 24.5 h, of slurries featuring different carbon content and (a) 0.5ME and (b) 3ME electrolytes. The inset reports the magnification of the plots at high frequencies. Data for the 0.5ME and 3ME electrolytes without carbon particles are also included.

Given that all the slurries exhibited a dynamic behavior, for a comparison of the electrical properties of samples featuring 0.5ME, 3ME, and 5ME electrolyte, we referred to the Nyquist plots collected after 24.5 h. The plots of 0.5ME- and 3 ME-based slurries are compared in Figure 8. Data obtained for the electrolytes 0.5ME and 3ME without carbon particles are also included. The Nyquist plots of 5ME-based slurries are reported in Figure S3a, b. In Figure 8, the high-frequency intercepts on the real axis of the plots for 0.5ME and 3ME without carbon provide the ionic resistance of the cells. For 0.5ME it was 623 Ω, lower than that for 3ME (673 Ω), in agreement with the higher conductivity of the former electrolyte with respect to that of the latter. On the contrary, the inset of Figure 8 shows that this trend does not hold for the carbonaceous slurries. Specifically, at a high carbon content, Rhf of PB054 (388 Ω) was higher than that of PB34 (335 Ω). This suggests that at high carbon content, the electronic percolation branch is more efficient in 3ME than in 0.5ME.

**Figure 9:**
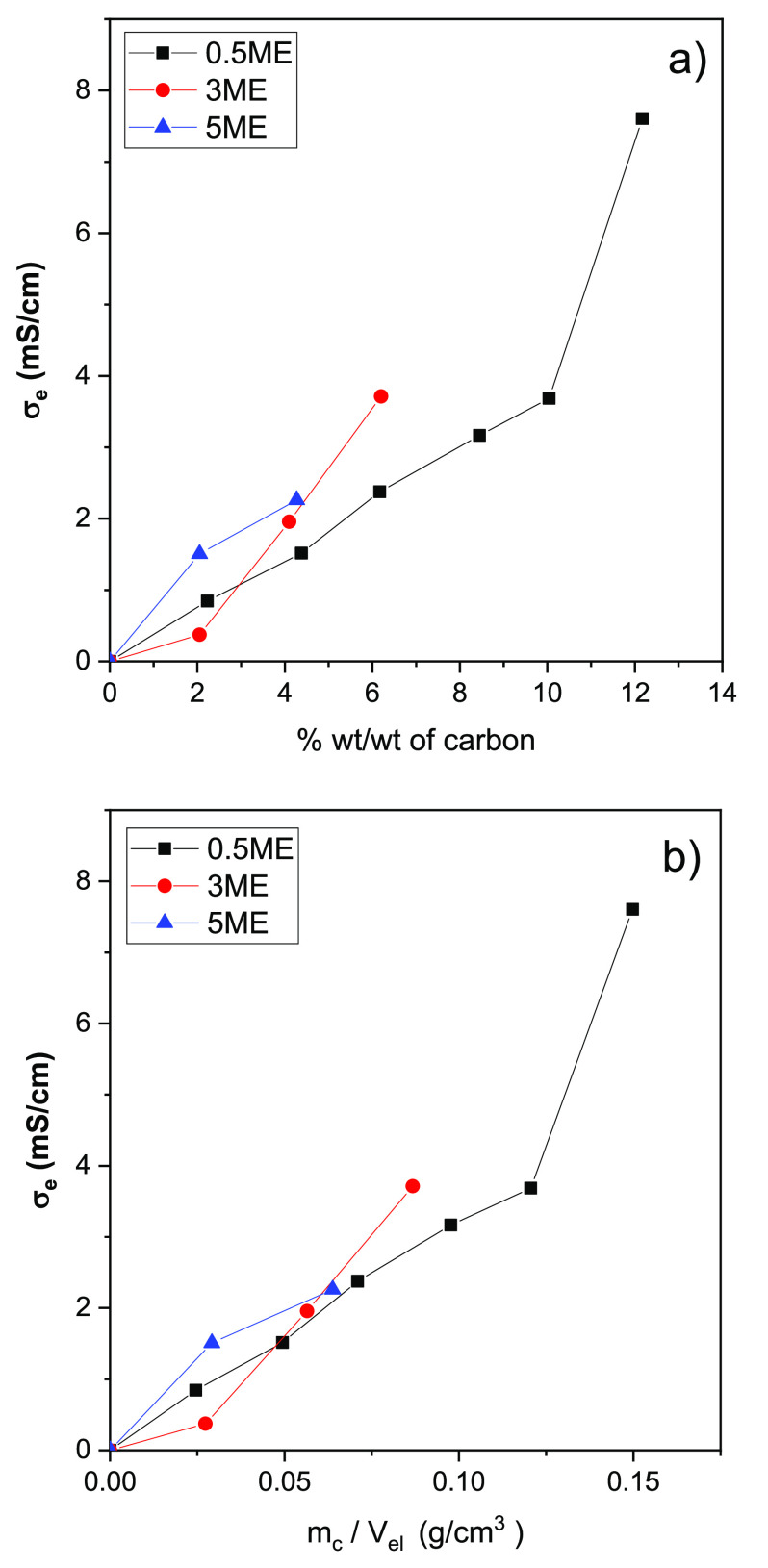
Electronic conductivity of 0.5ME- and 3ME-based slurries at different (a) mass percentages of carbon and (b) mass of carbon per electrolyte volume.

The electronic conductivity of the slurries σ_e_ was evaluated as described in section 2.4. The method enabled us to discriminate the electronic conduction provided by the carbon network from the ionic conduction provided by the electrolyte. Figure 9a shows the trend of σ_e_ vs carbon mass percentage for 0.5ME- and 3ME-based slurries. The electronic conductivity of the slurries featuring 0.5ME increases linearly up to 10% of carbon. In this range, σ_e_ varies from 0.8 mS cm^–1^ (2 wt %/wt) to 3.7 mS cm^–1^ (10 wt %/wt). In the range from 10% to 12 wt %/wt, a sharp increase in σ_e_ can be observed and the electronic conductivity reaches a value of 7.6 mS cm^–1^. Youssry et al.^17^ reported that the change of the slope of the σ_e_ vs carbon content plot marks the percolation threshold, i.e., the minimum amount of carbon that provides an efficient percolating network.^16^ Therefore, from Figure 9a, it is possible to evince that the percolation threshold of 0.5ME-based slurries corresponds to a carbon content of at least 10%.

Regarding 3ME-based slurries, σ_e_ linearly increases from 0.4 mS cm^–1^ at a carbon content of 2 wt %/wt to 3.7 mS cm^–1^ at 6 wt %/wt. Therefore, it is evident that with 3ME it is possible to achieve the same σ_e_ of 0.5ME but at a lower carbon mass percentage. For a deeper analysis, it should be considered that 0.5ME and 3ME have different densities, respectively of 1.08 and 1.31 g cm^–3^. Hence, 0.5ME- and 3ME-based slurries with the same carbon weight percentage feature a different mass of carbon per volume of solution. Figure 9b shows the trend of σ_e_ vs the values of carbon mass per volume, which are given for each sample in Table 1. In Figure 9b, the differences in conductivity of 0.5ME- and 3ME-based slurries are less marked than those observed in Figure 9a. However, still, the trend that distinguishes the 0.5ME- and 3ME-based slurries remains evident. Indeed, the σ_e_ value of 3.7 mS cm^–1^ is obtained at 0.08 g cm^–3^ with 3ME and at 0.12 g cm^–3^ with 0.5ME. This further confirms the positive effect of the higher ion concentration of 3ME vs 0.5ME on the electrical properties of the slurries. The high ionic strength of 3ME mitigates agglomeration and enhances a homogeneous dispersion of the carbon particles (see Figures 4 and 7). In turn, this facilitates the formation of an efficient percolating network and brings about a higher electrical conductivity with respect to slurries based on more diluted electrolytes like 0.5ME.

These findings are further supported by the electronic conductivity of the suspensions PB52 and PB54 that featured the highest LITFSI concentration (5 mol kg^–1^) and a carbon content equal to 2 and 4%, respectively. Indeed, for PB52 and PB54, σ_e_ was further improved and quantified as 1.50 and 2.26 mS cm^–1^. These values are included in Figure 9 and are higher than those featured by 0.5ME- and 3ME-based slurries at the same carbon content. The optical fluorescence images reported in Figure S4 highlight the good dispersion of carbon particles in the superconcentrated medium.

### 3.4 Rheological Properties of 0.5ME-, 3ME-, and 5ME-Based Slurries

**Figure 10:**
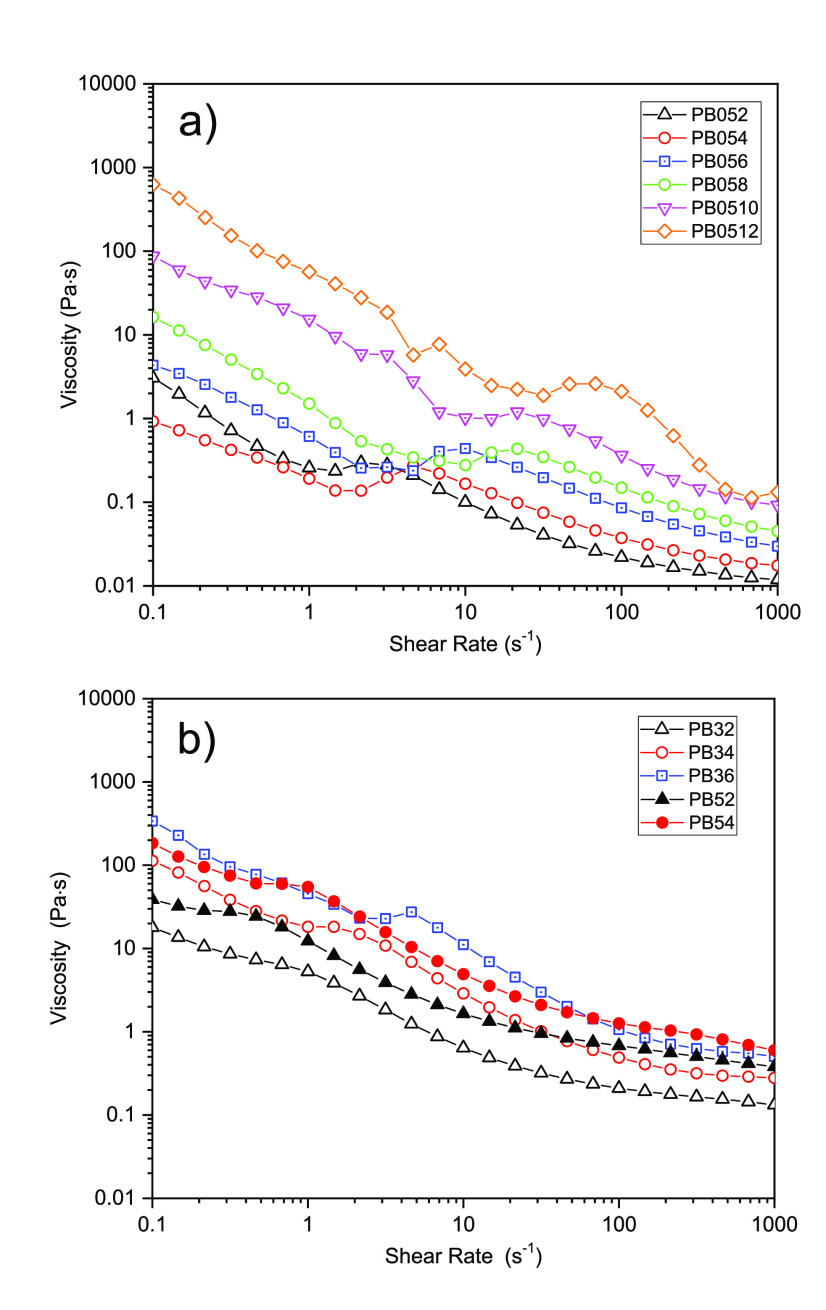
Viscosity response of (a) 0.5ME- and (b) 3ME- and 5ME-based slurry suspension on a shear rate range from 0.1 to 1000 s^–1^.

Rheological measurements were performed on 0.5ME-, 3ME-, and 5ME-based slurries to evaluate their viscosity at a shear rate ranging from 0.1 s^–1^ to 1000 s^–1^. Figure 10 shows the viscosity trends in relation with the shear rate applied. All the samples display a non-Newtonian behavior. Indeed, viscosity decreases by increasing the shear rate. Furthermore, the higher the carbon content of the slurry, the higher the viscosity of the samples. In fact, at 0.1 s^–1^, the viscosity increases from 3 Pa s for PB052 to 628 Pa s for PB0512, from 18 Pa s for PB32 to 338 Pa s for PB36, and from 38.7 Pa s for PB52 to 184 Pa s for PB54. A more detailed analysis of the plots highlights the presence of three regions for each curve. Viscosity decreases almost linearly at high and low shear rates, and a broad peak can be observed in between. This was also observed by Youssry et al.,^16,17^ who reported a three-phase behavior of the slurries: two share-thinning phases, at low and high share rates, separated by a transient shear-thickening phase located at the so-called critical shear rate point. This behavior was attributed to structural changes of the particles under Brownian and hydrodynamic forces that have a different impact on the slurry rheology at different shear rates. The shear thinning observed at low shear rates might be attributed to the breaking up of large agglomerates into smaller ones and to the reduction of the interparticle interactions. In turn, this brings about a lower resistance to the flow. At the critical shear rate point, a new transient phase is established. Here, small aggregates are rearranged as hydroclusters, shaped by the lubricating hydrodynamic forces. These hydroclusters are driven and sustained by the applied shear and contribute to the increase of the resistance to flow, therefore explaining the shear-thickening phase behavior. At the highest shear rates, the hydroclusters break into smaller particles that slide with weak resistance and set up a shear thinning phase. In Figure 10, it is evident that for all the samples, the shear-thickening phase shifts to higher shear rates as a consequence of the carbon content increase. At high carbon percentage, the initial clusters are strongly connected to each other, as shown by optical fluorescence images (Figures 4 and 7), and require a higher shear rate value to break up into smaller particles. It is worth noting that the shear thickening phases of 5ME- and 3ME-based slurries are less pronounced and slightly shifted to lower shear rates compared to 0.5ME samples, at the same amount of carbon content. This shift is caused by an easier disaggregation of the initial particles along with the formation of hydroclusters in superconcentrated electrolytes. In 3ME and, mainly, in 5ME, carbon particles are better distributed and their interactions are shielded by the high ionic strength of the fluids, as remarked by optical fluorescence images (Figure 7) and demonstrated by their efficient electronic percolation.

## 4 Conclusions

Semisolid RFBs, like those based on the lithium-ion battery chemistry, represent an emerging technology. Achieving an effective exploitation of the redox species dispersed in the anolyte and catholyte is a requisite. This can be attained by the optimization of the electrical percolating network that is typically realized by the use of carbon particles.

For the first time, to the best of our knowledge, the effect of the electrolyte ion concentration on the electronic percolating network of carbonaceous slurries for lithium-ion semisolid RFBs is here reported by a deep EIS analysis. EIS resulted to be a powerful technique to monitor the electrical properties of conductive slurries and their dynamic behavior. A model that describes the complexity and heterogeneity of the semisolid fluids by multiple conductive branches is proposed.

The study was carried out by taking into account different concentrations of LiTFSI in TEGDME, namely, 0.5 molal (0.5ME), 3 molal (3ME), and 5 molal (5ME) at different concentrations of carbon particles. The 0.5ME- and 3ME-based slurries featured a different dynamic behavior. For 0.5ME suspensions, the electronic component of the impedance decreased over time and the increase in carbon content accelerates the impedance evolution. For 3ME-based slurries, the dynamic behavior was different. The percolating resistance decreased at low carbon concentration but increased at the highest one. The EIS dynamic behavior has been explained with the sedimentation or agglomeration of carbon particles in the liquid phase that, in turn, brings about additional percolating branches with different electronic resistance with respect to fresh samples.

The main result of this work is that the electrical percolation is more efficient when the superconcentrated electrolyte is used. Indeed, the 3ME-slurries with 2 and 4% carbon feature percolating resistances that are almost 50 and 20% smaller than those of 0.5ME-based samples. This is related to a better carbon dispersion and connection in 3ME than in 0.5ME, achieved because of the high ionic strength of the superconcentrated solution that shields interparticle interactions. This was further supported by the results obtained with the 5ME-based slurries that featured the highest LITFSI concentration and electronic conductivities.

Therefore, this paper demonstrates an added advantage of superconcentrated electrolytes. Besides their recognized higher electrochemical stability with respect to a conventional solution, they enable a more stable and electronically conductive slurry. Both are key features for the development of semi-solid RFBs, even beyond lithium-ion battery chemistries.

## Supporting Information Available

Cell used for EIS measurements; original *R*_hf_ data for all the samples; additional EIS spectra of PB052, PB0512, PB52, and PB54 slurries; and optical fluorescence images of PB5-based slurries (PDF)

## Abbreviations

LiTFSI: lithium bis(trifluoromethane)sulfonamide
TEGDME: tetraethylene glycol dimethyl ether
EIS: electrochemical impedance spectroscopy
EES: electrical energy storage
RFBs: redox flow batteries
VRFBs: vanadium redox flow batteries
EFC: electrochemical flow capacitor
LCO: LiCoO_2_
LMNO: LiMn_1.5_Ni_0.5_O_4_
LTO: Li_4_Ti_5_O_12_
LFP: LiFePO_4_
0.5ME: 0.5 molal electrolyte
3ME and 5ME: 3 molal and 5 molal electrolyte
PB: Pureblack 315
*R*_e_: electronic resistance of the dispersed carbon particles
*R*_i_: ionic resistance of the electrolyte
*Q*_dl_: constant phase element for the electrical double-layer capacitance at the blocking electrode/slurry interfaces
σ_e_: slurry electronic conductivity
