# Supplementary material for: Improving the Electrical Percolating Network of Carbonaceous Slurries by Superconcentrated Electrolytes: An Electrochemical Impedance Spectroscopy Study
Source: ACS Appl Mater Interfaces. 2021 Mar 10;13(11):13872–82. doi: 10.1021/acsami.1c02439 (PMC8041255; doi:10.1021/acsami.1c02439)
Supplement: Supplementary file 1 — am1c02439_si_001.pdf [file am1c02439_si_001.pdf]

Supporting Information

Improving the electrical percolating network of carbonaceous slurries by super-concentrated electrolytes: an electrochemical impedance spectroscopy study

Alessandro Brilloni[1,2], Federico Poli[1,2], Giovanni Emanuele Spina[1], Damiano Genovese[1], Giorgia Pagnotta[1], Francesca Soavi[1,2,*]

[1] Department of Chemistry "Giacomo Ciamician", Alma Mater Studiorum Università di Bologna, Via Selmi 2, 40126 Bologna, Italy

[2] Bettery Srl , Via Pisacane 56, 74016 Massafra, Italy

* francesca.soavi@unibo.it

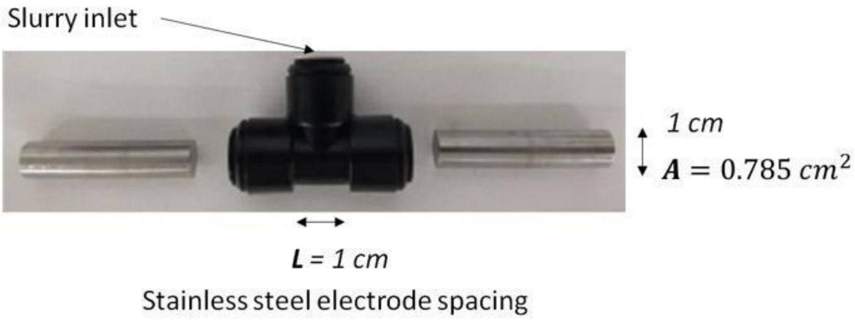

Figure S1 T-shaped cell used for the experiments

Table S1. Time evolution of R_{hf} values of 0.5ME-based slurries evaluated at 50 kHz

Measure	PB052	PB054	PB056	PB058	PB0510	PB0512
**-01**	486.30	395.6	323.64	266.70	252.70	153.15
**-02**	483.20	396.2	323.64	265.00	253.00	152.90
**-03**	475.80	391	319.90	271.00	251.70	152.20
**-04**	473.90	388	270.50	256.71	234.10	140.00

Table S2. Time evolution of R_{hf} values of 3ME-based slurries evaluated at 50 kHz

Measure	PB32	PB34	PB36
**-01**	559	337.7	255.65
**-02**	557	338.9	255.9
**-03**	543	340.9	258.6
**-04**	546	335.4	245.7

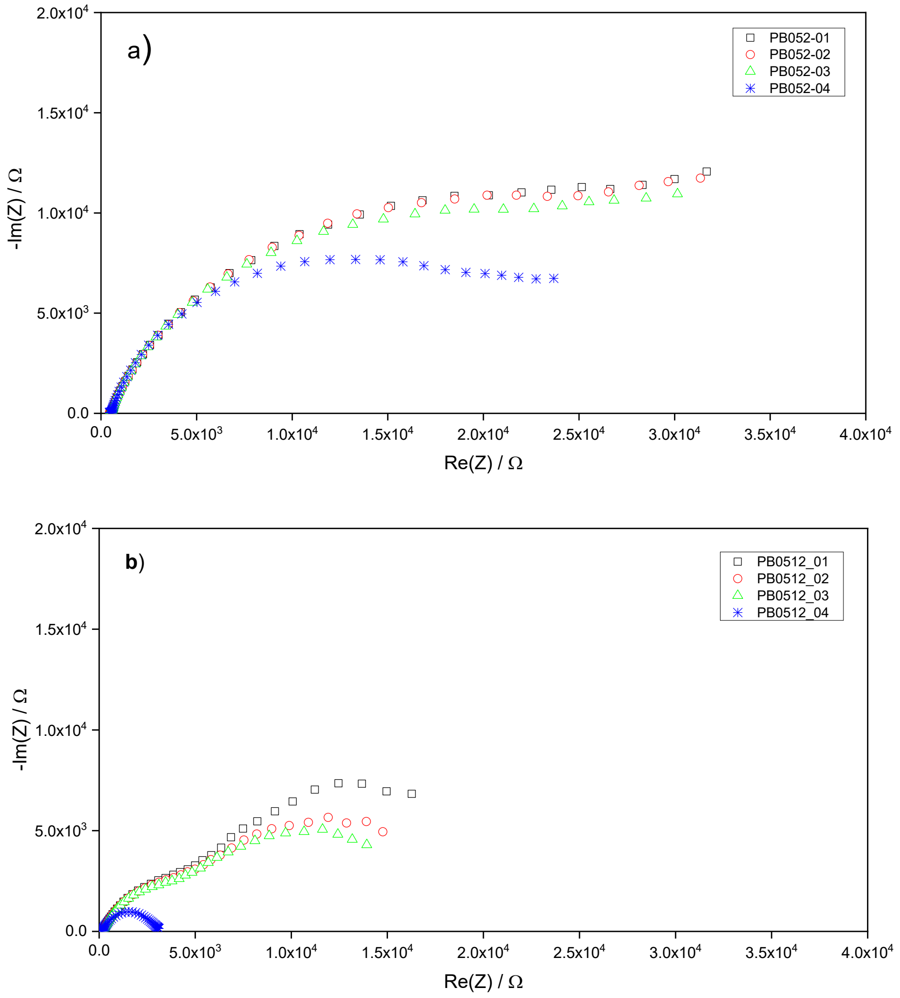

Figure S2. Nyquist plot evolution over time of 0.5ME-based slurries with a) 2% of carbon and b) 12% of carbon at frequency range of 200kHz–100 mHz.

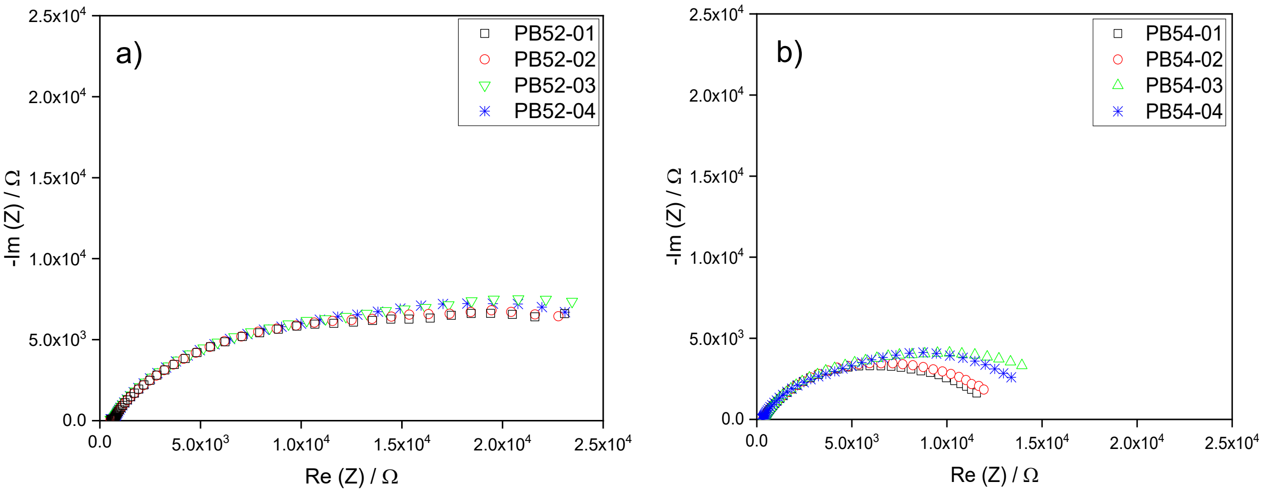

Figure S3 Nyquist plots evolution over time of 5ME-based slurries with different carbon percentages of a) 2%, b) 4% from 200kHz to 100 mHz.

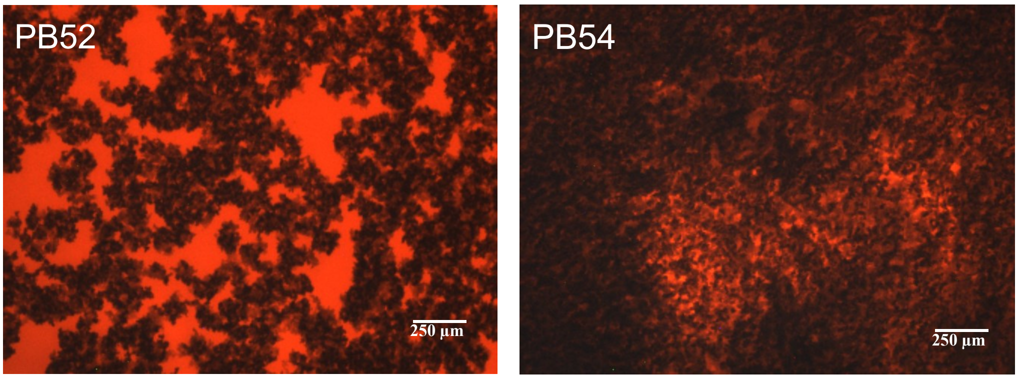

Figure S4: Optical fluorescence images of 5ME based slurries with different carbon percentage.
